# Resource Allocation for Epidemic Control Across Multiple Sub-populations

**DOI:** 10.1007/s11538-019-00584-2

**Published:** 2019-02-26

**Authors:** Ciara E. Dangerfield, Martin Vyska, Christopher A. Gilligan

**Affiliations:** Department of Plant Sciences, University of Cambridge, Downing Street, Cambridge, CB2 3EA UK

**Keywords:** Epidemiological modelling, Optimal control of epidemics, Metapopulation model

## Abstract

The number of pathogenic threats to plant, animal and human health is increasing. Controlling the spread of such threats is costly and often resources are limited. A key challenge facing decision makers is how to allocate resources to control the different threats in order to achieve the least amount of damage from the collective impact. In this paper we consider the allocation of limited resources across *n* independent target populations to treat pathogens whose spread is modelled using the susceptible–infected–susceptible model. Using mathematical analysis of the systems dynamics, we show that for effective disease control, with a limited budget, treatment should be focused on a subset of populations, rather than attempting to treat all populations less intensively. The choice of populations to treat can be approximated by a knapsack-type problem. We show that the knapsack closely approximates the exact optimum and greatly outperforms a number of simpler strategies. A key advantage of the knapsack approximation is that it provides insight into the way in which the economic and epidemiological dynamics affect the optimal allocation of resources. In particular using the knapsack approximation to apportion control takes into account two important aspects of the dynamics: the indirect interaction between the populations due to the shared pool of limited resources and the dependence on the initial conditions.

## Introduction

The infection burden of many epidemics outstrips the resources available to treat all individuals (Lipsitch et al. 2000; Kiszewski et al. 2007). Furthermore, characteristics of disease spread may differ between different groups of the populations. The challenge facing central decision makers who seek to control an epidemic at the landscape scale is therefore how to allocate limited resources in order to minimise the impacts of disease across the entire population? Optimising the deployment of control requires consideration of both epidemic dynamics and economic factors, including the costs of the epidemic and control as well as budgetary constraints and availability of resources.

Previous studies have used control theory to determine the optimal allocation of limited resources to minimise the impacts from an epidemic (Rowthorn et al. 2009; Ndeffo Mbah and Gilligan 2011; Zaric and Brandeau 2001a, b; Brandeau et al. 2003; Hansen and Day 2011; Zhou et al. 2014). For simplicity, many early studies considered the application of a single control within a single target population (Hansen and Day 2011; Zhou et al. 2014). However, heterogeneities in the host population are known to be important in the invasion and persistence of human, animal and plant pathogens (Ferguson et al. 2001; Dye and Gay 2003; Stacey et al. 2004). Within human populations heterogeneities arise, for example through different contact patterns amongst sub-populations (Wallinga et al. 1999). For animal and plant pathogens, it is often the spatial structure that is critical in the invasion and persistence of the pathogen (Ferguson et al. 2001; Stacey et al. 2004; Keeling et al. 2001). Such heterogeneities in the characteristics related to epidemic spread amongst sub-populations of the host population are typically captured using structured metapopulations (Grenfell and Bolker 1998, 2000). Rowthorn et al. (2009) consider the optimal deployment of limited resources across two different but interconnected regions of equal size. Minimising the discounted number of infected individuals over a fixed time horizon within the susceptible–infected–susceptible (SIS) model, Rowthorn et al. (2009) find an arguably counterintuitive result that treatment should be preferentially directed at the sub-population with the lowest number of infected individuals. The inclusion of temporary immunity, essentially extending from an SIS to an SIRS model, alters the optimal strategy whereby it is initially optimal to preferentially treat the more infected sub-population and then switch to treating the less infected sub-population (Ndeffo Mbah and Gilligan 2011). The limitation of the studies by Rowthorn et al. (2009) and Ndeffo Mbah and Gilligan (2011) is that they only consider two sub-populations, but in reality a larger number of sub-populations is often needed to capture the heterogeneities within a target population. A key goal of the current work is to extend the work of Rowthorn et al. (2009); Ndeffo Mbah and Gilligan (2011) to the problem of $n\ge 2$ sub-populations.

Work on the allocation of resources between two sub-populations uses an optimisation approach based on the Hamiltonian method (Rowthorn et al. 2009) and the Pontryagin maximum principle (Ndeffo Mbah and Gilligan 2011), which provides analytic insight into the form of the optimal allocation strategy. Extending this approach to the general problem of *n* populations leads to a large number of equations that cannot be solved analytically. Indeed, Zaric and Brandeau (2001b) show that the general problem of the allocation of limited resource across *n* coupled sub-populations is intractable. Therefore, numerical techniques are typically used to solve such problems as in Richter et al. (1999), Zaric and Brandeau (2001a), Zaric and Brandeau (2001b) and Brandeau et al. (2003). However, numerical approaches lose the intuitive insight that analytic approaches provide about underlying mechanisms. The loss of intuitive insight limits the use of optimal control methods in determining generalisable rules and simple heuristics that can be used by decision makers. Indeed, Brandeau et al. (2003) identify the need for simple, easy to use guidelines based on the optimal solution in order to make practical use of optimal control theory by decision makers. The challenge therefore is how to generalise the results from Rowthorn et al. (2009) and Ndeffo Mbah and Gilligan (2011) to the case of $n\ge 2$ sub-populations.

The primary goal of this paper is accordingly to provide insight into the general form of the optimal allocation of treatment across *n* sub-populations when the resources available for control are limited. In particular, we seek to answer the following questions:

- How do epidemiological dynamics and economic constraints impact the optimal allocation of resources across *n* sub-populations?
- How does the indirect interaction between sub-populations that arises from the limited availability of resources affect the optimal allocation of resources to the different sub-populations?
- Can we develop a simple easy-to-use allocation strategy that is close to the optimal solution?

We consider a simplified model of an epidemic that directly incorporates the economic constraint and we consider the final size of the epidemic as the measure of impact. We are therefore able to use understanding of the long-term dynamics of the model in order to gain analytic insight into the form of the optimal strategy without the use of optimal control theory, which becomes intractable for n-dimensional problems such as that studied in this paper. Some options for relaxing the assumptions to analyse more realistic epidemic scenarios are addressed in the discussion.

## Methods

### Model

Forests often contain a number of different tree species, and in recent years there has been a move towards mixed-species in order to make forests more resilient to disturbances and stresses such as those posed by climate change (Kerr et al. 2015). To understand how to allocate resources optimally in order to minimise the impacts of multiple threats across different tree species is challenging using traditional optimal control theory approaches. The problem quickly becomes intractable for the general n-dimensional problem when n greater than 2.

We consider the optimal control of epidemics in *n* sub-populations, each of size $N_{i}$. Each population is considered to be a different tree species within a forest under threat from multiple different pests or pathogens. This is a common problem facing many non-commercial forests which are typically composed of a large number of different tree species under threat from a number of invasive species (Baker et al. 2014). A pest or pathogen is typically specialised to a given tree species, for example the ash dieback fungus only infects ash trees and Dothistroma needle blight only affects pine trees. Therefore, we assume that infection can only be transmitted within a sub-population (species) and not between sub-populations. This means the sub-populations are independent, which allows us to reduce the optimal control problem to study the dynamics of the individual sub-populations.

We assume that each epidemic can be described by a susceptible–infected–susceptible (SIS) compartmental framework since it is a very general epidemic model applicable to a wide number of different pathogens (Anderson and May 1991). The SIS model assumes individuals return to the susceptible compartment following natural recovery or treatment. It is therefore characteristic of infections, such as gonorrhoea or Dothistroma, where recovered individuals do not gain immunity (Anderson and May 1991). We consider a treatment that increases the rate of recovery of infected hosts by a fixed amount, $\eta _i$ (Rowthorn et al. 2009; Ndeffo Mbah and Gilligan 2011). For tree diseases, examples of such treatments are application of pesticides or fungicides directly to the tree (Masoa et al. 2014). Such control options are common especially when aerial spraying is banned, as in the UK, and felling is a less popular option with the general public (Sheremet et al. 2017). In human and animal health, examples of such a treatment could be antibiotics. We assume that the rate of recovery due to treatment is different for each sub-population since the efficacy for a given pesticide/fungicide is likely to vary for across tree species.

To model the economic constraint, we assume that the control resources are limited and no more than a proportion, $\gamma _i$, of the hosts within sub-population *i* can be treated at any given time. The dynamics of the proportion of infected hosts in sub-population *i* ($I_i$) is therefore given by the following equation

1 $$\begin{aligned} \frac{\mathrm{d}I_i}{\mathrm{d}t} = \beta _i I_i(1-I_i) - I_i - \eta _i \min (I_i, \gamma _i), \end{aligned}$$

where $\beta _i I_i$ is the rate at which a susceptible host in sub-population *i* gets infected (assuming homogeneous mixing within sub-population *i*). Note that time is scaled so that one unit of time corresponds to the average length of one infectious period in the absence of treatment, which is equivalent to setting the recovery rate, $\mu _i$ to be $\mu _i=1$ in the standard SIS model. We note that the time scaling in each sub-population will be different. However, since infection cannot be transmitted between sub-populations and we only consider the dynamics of the system once equilibrium has been reached this does not affect our analysis. We allow the transmission parameter $\beta _i$ and the efficacy of the treatment $\eta _i$ to vary amongst sub-populations. This generality captures the fact that epidemiological dynamics are likely to vary across sub-populations which in our example arises because of differences in pathogen characteristics for distinct tree species. Finally we assume that the initial level of infection at time $t=0$, $I_i(0)$, varies across the sub-populations.

We assume resources are allocated to sub-populations at time $t=0$ and cannot be reallocated later, which is applicable if reallocation is expensive, for example when decisions are taken by central planners (Zaric and Brandeau 2001a, b; Brandeau et al. 2003). We allow the cost of treatment per host (i.e. amount of resource per host treated), $k_i$, to vary amongst sub-populations. For example, it may be harder to administer treatment to certain sub-populations. We assume there is a limited amount, *M* units, of the resource available. Even though there is no direct coupling, the assumption of a limited shared resource pool gives rise to an indirect interaction between sub-populations. When more resources are allocated to one of the sub-populations, the disease prevalence in that sub-population decreases, but there is consequently less resource for allocation into the other sub-populations. The proportions of infected individuals in the sub-populations are therefore anti-correlated.

Consider an allocation strategy that allocates $x_iM$ resource into sub-population *i*, where $x_i\in [0, 1]$ is the proportion of resource allocated to sub-population *i* and so $\sum _i x_i = 1$. The proportion of individuals that can be treated in sub-population *i* is $\gamma _i = x_iM/(N_i k_i)$. If there is enough resource, then all infected individuals are treated. Otherwise, treatment is allocated in order to minimise the long-term level of infection. Therefore, we seek to choose an allocation strategy that puts a proportion, $x_i$, of the total resource into sub- population *i*, in order to minimise the objective function given by

2 $$\begin{aligned} J(x_i) = \sum _i N_i I_i^{\infty }(x_i), \end{aligned}$$

subject to the resource constraint

3 $$\begin{aligned} \sum _i k_i N_i \gamma _i \le M, \end{aligned}$$

where $I_i^{\infty }(x_i)$ is the equilibrium level of infection that is reached if the proportion of resource allocated to sub-population *i* is $x_i$.

The objective function in Eq. (2) is natural and easy to understand. Mathematically, it is, in fact, a special case of a more general, commonly used (Rowthorn et al. 2009; Ndeffo Mbah and Gilligan 2011) objective function defined as the average over some time interval (0, *T*) of the number of the infected individuals,

4 $$\begin{aligned} J = \frac{1}{T}\sum _i N_i\int _0^T I_i(t) \mathrm{d}t. \end{aligned}$$

The objective function we consider here can be interpreted as the average over a time interval (0, *T*) when the time horizon *T* becomes very large, in the limit $T\rightarrow \infty$. Other potential choices for the objective function would be to use an equivalent of quality adjusted life years (QALYs) as in (Zaric and Brandeau 2001a; Brandeau et al. 2003) or the infections averted as in Zaric and Brandeau (2001b) as a measure of the impact of the infection. The advantage of the choice of objective function in Eq. 2 is the following. Firstly, it is applicable to epidemics within humans, animals and plants, while QALYs are a measure specific to human health. Secondly, since the control we consider increases the recovery time, which essentially reduces the infection burden, it makes more sense to consider the impact of control as the reduction in infection burden rather than infections averted, which is more appropriate for control measures that reduce the transmission rate. Thirdly, taking the infinite time horizon limit is appropriate for our motivating example of multithreats within a forest since typically the time scales of interest for preserving a forest are very long. Finally, considering the average level of infection over a long time horizon allows us to use analysis of the equilibrium dynamics of the model to characterise the optimal solution, rather than using formal optimal control theory, which is intractable for the general problem of n-sub-populations.

The equilibrium level of infection in the absence of treatment for sub-population *i* is $1-1/\beta _{i}$ which we denote by $C^{(i)}_{0}$. When there are sufficient resources to treat all infected hosts, that is $I_{i}<\gamma _{i}$, then the equilibrium level of infection, which we term the full treatment equilibrium ($C_T^{(i)}$), is

5 $$\begin{aligned} C_T^{(i)}=1-(1+\eta _{i})/\beta _i, \end{aligned}$$

(see “Appendix A” for details). We refer to a sub-population as *saturated* whenever the resources allocated to it cause the long-term prevalence to be $C_{T}^{(i)}$. Therefore saturation is the state in which adding more resources no longer has an effect on the objective function. The model parameters and variables along with baseline parameter values used in simulations are summarised in Table  1.

**Table 1:** Table showing the parameters and variables used, together with their descriptions

Parameter/variable	Description
$\beta _i$	Rate of infection in population *i*
$\eta _i$	Additional recovery rate provided by the treatment
$C_T^{(i)}$	Endemic disease prevalence given full treatment in population *i*, $1-\frac{1+\eta _i}{\beta _i}$
$C_0^{(i)}$	Endemic disease prevalence given no treatment in sub-population *i*, $1 - \frac{1}{\beta _i}$
$\gamma _c^{(i)}$	Proportion of hosts in sub-population *i* that require simultaneous treatment necessary for saturation
$N_i$	Size of the $i\text {th}$ sub-population
*M*	The maximum amount of resources available
$x_i$	Proportion of the resource that is allocated to the sub-population *i*
$k_i$	The cost of treating one host in sub-population *i*
*n*	Number of sub-populations

The most used derived parameters are also included, for convenience

### Simple Allocation Strategies

In Sect. 2.3 we derive a simple heuristic that closely approximates the optimal solution but which is more intuitive. We compare the performance of this heuristic with four straightforward allocation strategies that a decision maker might use. All strategies are compared with the performance of the exact optimal which is calculated using a ‘brute force’ numerical approach (see Sect. 2.4 for details). The simple allocation strategies we consider are:

- *Proportional allocation* The amount of resource allocated to the $i\text {th}$ sub-population is proportional to the size of the sub-population, $N_i$, so the proportion of resource allocated to sub-population *i* is
  6 $$\begin{aligned} x_i= \frac{N_i}{\sum _i N_{i}}. \end{aligned}$$
- *Equal allocation* The same amount of resource is allocated to each of the sub-populations, so the proportion of resource allocated to sub-population *i* is $x_i = 1/n$.
- *Allocate to the largest strategy* We look at which sub-population we need to saturate to achieve the greatest decrease in the objective function and then repeat until we cannot saturate anymore, at which point resources are allocated to the remaining sub-population that would result in the greatest decrease in the objective function. In the case when both the epidemiological and the cost parameters are identical across all sub-populations, this is equivalent to saturating each sub-population in order of size from largest to smallest, hence we refer to this as the “allocate to the largest” strategy.
- *Allocate to the smallest strategy* This strategy is the opposite of the allocate to the largest strategy, in that we saturate the sub-population that leads to the smallest decrease in the objective function and then repeat until we cannot saturate any more, at which point we allocate the remaining resource to the sub-population that would give the smallest decrease in the objective function. This strategy is equivalent to saturating each sub-population in order of size from smallest to largest when both the epidemiological and cost parameters are identical. Hence we refer to this as the “allocate to the smallest” strategy.

The proportional and equal allocation strategies can be considered more socially equitable from the perspective of the chance of every individual receiving treatment, as compared with the allocate to largest and the smallest strategies, and potentially to the optimal solution. Similar strategies to the ones above were previously considered in Ndeffo Mbah and Gilligan (2011), although we note that they are not identical since Ndeffo Mbah and Gilligan (2011) allow for reallocation of resources.

### Model Analysis

In this section we show that the optimal allocation using analysis of the fixed points is as follows: saturate some subset *S* of the sub-populations such that no further sub-populations can be saturated and then allocate all the resources left over into one of the remaining unsaturated sub-populations. Therefore, the optimal strategy lies on the boundary of possible allocation strategies and no interior solution to the problem exists. Furthermore, we determine a simple heuristic to determine which sub-populations should be saturated. Since the optimal solution involves the saturation of sub-populations, we begin by considering the minimum amount of resource needed to saturate a sub-population that depends on the long-term dynamics of the system.

#### Minimal Treatment to Saturate a Sub-population

We begin by considering the minimum amount of resource, $\gamma _C^{(i)}$, necessary to ensure that sub-population *i* ends up in the full treatment equilibrium, that is $I^{(i)}\rightarrow C_{T}^{(i)}$ as $t\rightarrow \infty$. This depends on the dynamics of the system at equilibrium, which are analysed in detail in “Appendix A”. In particular, the dynamics differ in two distinct regions of parameter space, depending on the epidemiological parameters for the rate of infection, $\beta _i$, and the rate of recovery following treatment $\eta _i$.

When $\eta _i<(\beta _i-1)/2$, $C_{T}^{(i)}$ is the only stable equilibrium (see “Appendix A” for details) and so the minimum amount of treatment required is simply $\gamma _C^{(i)}=C_T^{(i)}$. When $\eta >(\beta -1)/2$, allocating $\gamma _{C}^{(i)}=C_{T}^{(i)}$ resources may no longer be sufficient to ensure the full treatment endemic equilibrium $C_{T}^{(i)}$ is reached (i.e. that the population is saturated). This is because in this region of parameter space there exist three equilibria, $C_{T}^{(i)}$, $I_A^{(i)}$ and $I_B^{(i)}$ (formulae for $I_A^{(i)}$ and $I_B^{(i)}$ are given in “Appendix A”). In particular, $C_{T}^{(i)}$ and $I_B^{(i)}$ are both stable ($C_{T}^{(i)}<I_B^{(i)}$) and they are separated by the unstable equilibrium $I_A^{(i)}$. In this case we use the analysis of the system dynamics at equilibrium, given in “Appendix A”, to determine the minimum amount of treatment required to ensure $I_{i}^{\infty }=C_{T}^{(i)}$ (i.e. to ensure the sub-population is saturated), based on the initial prevalence of infection in the sub-population, $I_{0}^{(i)}$. If the initial prevalence $I_0^{(i)}$ satisfies $I_0^{(i)}<C_T^{(i)}$, then we only need to allocate $\gamma _C^{(i)}=C_T^{(i)}$. When the initial condition satisfies $C_T^{(i)}< I_0^{(i)} < C_0^{(i)}/2$, the necessary $\gamma _{C}^{(i)}$ is given by the intersection of the line $I=I_0^{(i)}$ with the curve $I=I_A^{(i)}$, that is, it is the solution to the equation

7 $$\begin{aligned} \frac{1}{2}\left( C_0^{(i)} - \sqrt{\left( C_0^{(i)}\right) ^2 - \frac{4\eta _i\gamma _i}{\beta _i}}\right) = I_0^{(i)} \end{aligned}$$

which is given by $\gamma _C^{(i)} = \frac{\beta _i}{\eta _i}I_0^{(i)}(C_0^{(i)} - I_0^{(i)})$. Finally if the initial prevalence satisfies $I_0^{(i)}>C_0^{(i)}/2$, that is the initial prevalence is greater than the $I_A^{(i)}$ at the point where equilibria $I_A^{(i)}$ and $I_B^{(i)}$ annihilate (“Appendix A”) then we need to allocate $\gamma _{c}^{(i)}=\frac{\beta (C_0^{(i)})^2}{4\eta _i}$.

We summarise the conditions and formula for the minimum amount of treatment for the different possible scenarios in Table 2.

**Table 2:** Table giving the parameter regimes, conditions on the initial conditions and the subsequent formulas for the minimal amount of treatment required to saturate sub-population *i*

Parameter region	Initial condition criterion	Allocation
$\eta _i<(\beta _i-1)/2$	all $I_0^{(i)}$	$\gamma _C^{(i)}=C_T^{(i)}$
$\eta _i>(\beta _i-1)/2$	$I_0^{(i)}<C_T^{(i)}$	$\gamma _C^{(i)}=C_T^{(i)}$
$\eta _i>(\beta _i-1)/2$	$C_T^{(i)}<I_0^{(i)}<=C_0^{(i)}/2$	$\gamma _c^{(i)} = \frac{\beta _i}{\eta _i}I_0^{(i)}(C_0^{(i)} - I_0^{(i)})$
$\eta _i>(\beta _i-1)/2$	$C_0^{(i)}/2<I_0^{(i)}$	$\gamma _c^{(i)} = \frac{\beta _i(C_{0}^{(i)})^2}{4\eta _i}$

The analysis shows dependence of the minimum amount of treatment required to saturate a sub-population on the initial conditions. This dependence on initial conditions arises due to the dynamics of the SIS model in the presence of an economic constraint on available treatment resources. Therefore, even if all sub-populations are identical in terms of the epidemiological and economic parameters, different amounts of resource may be needed to saturate each sub-population, depending on the initial level of infection within a given sub-population. Since resources are limited, differences in the amount of resource required to saturate a sub-population are important in determining the optimal allocation of resources.

#### The Optimal Strategy is to Saturate a Subset of Sub-populations

Let $S\subset \lbrace 1,\ldots ,n\rbrace$ be the subset of sub-populations that are saturated under the allocation $\lbrace x_i\rbrace$. Then the objective function to be minimised, Eq. (2), can be written as follows

8 $$\begin{aligned} J&= \sum _i N_i I_i^{\infty }(x_i) \end{aligned}$$

9 $$\begin{aligned}&= \sum _{i\in S} C_T^{(i)} N_i + \sum _{i\not \in S}\frac{N_i}{2}\left( C_0^{(i)} + \sqrt{\left( C^{(i)}_0\right) ^2 - \frac{4\eta _i M x_i}{\beta _i N_i k_i}}\right) . \end{aligned}$$

In the first term in Eq. (9), each sub-population is saturated and so the endemic infection level will be $C_T^{(i)}$, by definition. In the second term none of the populations are saturated and so the endemic level of infection is given by the equilibrium in the case when there is insufficient resource to treat all infected individuals. The form of this equilibrium, given by the term in the second summation in Eq. (9), is found by fixed point analysis of the model for epidemic dynamics in the presence of control given by Eq. (1) in the case where $I_{i}>\gamma _i$. See “Appendix A” for details.

We begin by showing that there is only one interior local extremum and it cannot be a minimum. Consider an allocation strategy $x_i$ which does not saturate any of the populations. The objective function is given by

10 $$\begin{aligned} J(x_1,\ldots , x_{n-1}) = \sum _i^n \frac{N_i}{2}\left( C_0^{(i)}+\sqrt{\left( C_0^{(i)}\right) ^2 - \frac{4\eta _i M x_i}{\beta _i N_i k_i}}\right) , \end{aligned}$$

where $x_n = 1-x_1-\cdots -x_{n-1}$. To find the local extrema, we solve the set of equations $\partial J/\partial x_i = 0$. This yields that for each *i*,

11 $$\begin{aligned} \frac{\beta _i k_i}{\eta _i}\sqrt{\left( C_0^{(i)}\right) ^2 - \frac{4\eta _i M}{\beta _i k_i N_i}x_i} = \text {constant}. \end{aligned}$$

To find the constant and hence the unique local extreme, $x^\star$, we use the condition $\sum _i^n x^\star _i = 1$. This gives

12 $$\begin{aligned} x^\star _i = a_i - \frac{\sum _j a_j - 1}{\sum _j b_j}b_i, \end{aligned}$$

where

13 $$\begin{aligned}&a_i = \frac{N_i \left( C_0^{(i)}\right) ^2 \beta _i k_i}{4\eta _i M}, \end{aligned}$$

14 $$\begin{aligned}&b_i = \frac{\eta _i N_i}{4M\beta _i k_i}. \end{aligned}$$

To show that this point cannot be a local minimum and therefore cannot be the solution to the optimisation problem, we need to prove that the $n-1\times n-1$ matrix of the second derivatives $DJ^\star$ has at least one negative eigenvalue, (Arrowsmith and Place 1992). Differentiating *J* twice yields

15 $$\begin{aligned}&DJ^\star _{ii} = -\frac{2 M^2}{S^{3/2}}\left( \frac{\beta _n k_n}{\eta _n N_n} + \frac{\beta _i k_i}{\eta _i N_i}\right) \end{aligned}$$

16 $$\begin{aligned}&DJ^\star _{i\not = j} = -\frac{2 M^2}{S^{3/2}}\frac{\beta _n k_n}{\eta _n N_n}. \end{aligned}$$

This is a real, symmetric matrix with negative entries and therefore it has a negative eigenvalue (Theorem, “Appendix B”). The result implies that the point $x^\star$ cannot be a local minimum. Since this is the only local extremal point of the system, the allocation strategy minimising *J* must saturate at least one of the sub-populations. Suppose the optimum lies in the subspace of all possible strategies where sub-population *m* is saturated. The resources remaining after saturating *m* are $M - N_m k_m\gamma _c^{(m)}$ (where $\gamma _c^{(m)}$ are the resources needed to saturate sub-population *m*). After we saturate *m*, the problem is to minimise *J* in the remaining sub-populations given the unused resources. This is qualitatively the same as before, and so the solution must saturate at least one of the remaining sub-populations. This argument can be repeated until there are insufficient resources to keep saturating.

We have thus shown that the optimal allocation must lie on the boundary of the surface that defines the potential optimal strategies, that is we need to saturate sub-populations until further saturation is not possible. A key question therefore is how should the remaining resources be distributed amongst those sub-populations that are not saturated?

The question of where to allocate the remaining resources is the same as the problem of where to allocate resources if we cannot saturate any of the sub-populations. Suppose that in the optimal allocation, some subset *X* of the sub-populations share the resources, that is more than one sub-population has nonzero amount of resources allocated to it. There is no coupling between the sub-populations and so we can consider the subset *X* in isolation. Since none of the sub-populations in *X* are saturated and none have zero resources allocated to it, as far as *X* is concerned, this allocation is an interior one. We proved above however, that there can be no interior local minimum of any number of sub-populations. Therefore, when no sub-populations can be saturated, all the resources should be allocated to a single sub-population, that is $$\gamma _m = M/(k_m N_m)$$ for some *m* and $$\gamma _i = 0$$$$\forall i\not = m$$.

The above analysis shows that the optimal allocation strategy is as follows: saturate some subset *S* of sub-populations such that no further saturating is possible and then allocate all the unused resources into one of the remaining unsaturated sub-populations. The optimal strategy raises two main questions:

1. How should the subset of sub-populations that are saturated with treatment, *S*, be chosen?
2. Into which sub-population should the remaining resource be allocated?

#### Which Sub-populations Should Receive Treatment?

We consider the first question, that is, if we can saturate some of the sub-populations, which should we pick? Suppose a set *S* of the sub-populations is saturated, so $S = \lbrace i\vert i\text { is saturated}\rbrace$. Mathematically, we want to know which choice of *S* minimises the objective function. We refer to the set of the remaining unsaturated sub-populations as *R*, so $R=\lbrace i\vert i\not \in S\rbrace$. The resource left after saturating *S*, $M_R$, is given by

17 $$\begin{aligned} M_R = M - \sum _{i\in S}N_i k_i\gamma _c^{(i)}. \end{aligned}$$

The restriction on *S* that no more sub-populations can be saturated can be put in mathematical terms as

18 $$\begin{aligned} M_R < \gamma _c^{(i)}N_i k_i,\quad \forall i\in R. \end{aligned}$$

The objective function, as a function of *S*, is given by

19 $$\begin{aligned} J(S)&= \sum _{i\in S}\ C_T^{(i)} N_i+\sum _{i\in R\setminus \lbrace m_0\rbrace } C_0^{(i)} N_i \nonumber \\&\quad + \frac{1}{2}N_{m_0}\left( C_0^{(m_0)} +\sqrt{\left( C_0^{(m_0)}\right) ^2 - \frac{4\eta _{m_0} M_R}{\beta _{m_0} N_{m_0}k_{m_0}}}\right) , \end{aligned}$$

where $m_0$ is the index of the sub-population receiving the resources left after saturation. The task is to minimise *J*(*S*) by an appropriate choice of *S*. This is a difficult problem, mainly because it is hard to capture how $m_0$ depends on the choice of the set *S*.

We approach the optimisation problem given by Eq. (19) by considering an approximation where we neglect the term $4\eta _{m_0} M_R/\beta _{m_0} N_{m_0} k_{m_0}$. This amounts to ignoring the resources that are left over after saturating the sub-populations in *S* when selecting the optimal *S*. The error in the objective function arising from this approximation is at most $\frac{1}{2}C_0^{m_0} N_{m_0}$. The objective function as a function of *S* can then be written as

20 $$\begin{aligned} J(S)&= \sum _{i\in S} C_T^{(i)} N_i + \sum _{i\not \in S} C_0^{(i)} N_i \end{aligned}$$

21 $$\begin{aligned}&= \sum _i^n C_0^{(i)} N_i - \sum _{i\in S}(C_0^{(i)} - C_T^{(i)})N_i. \end{aligned}$$

To minimise *J*(*S*) we need to maximise $\sum _{i\in S}(C_0^{(i)} - C_T^{(i)})N_i$. In other words we need to saturate a set *S* such that the value of host saved by treatment is as large as possible, given the constraint on available resources. The problem can be rephrased as follows:

22 $$\begin{aligned}&\text {Find subset }S\text { that maximises}\quad \sum _{i\in S}\left( C_0^{(i)} - C_T^{(i)}\right) N_i \end{aligned}$$

23 $$\begin{aligned}&\text {Subject to the constraint}\quad \sum _{i\in S}k_i\gamma _C^{(i)}N_i < M, \end{aligned}$$

where $\gamma _{C}^{(i)}$ is the minimal amount of treatment required to saturate sub-population *i*, which will depend on the initial conditions when $\eta _i>(\beta _i-1)/2$. Formulae for $\gamma _{C}^{(i)}$ are given in Table 2.

Equation (23) is a variation on the knapsack problem, well known in computer science (Martello and Toth 1990; Skiena 1999; Kellerer et al. 2004). Its uses are wide ranging, for example finding the optimal way to cut raw materials (Kellerer et al. 2004), construction of investment portfolios (Kellerer et al. 2004) and the construction and scoring of tests (Feuerman and Weiss 1973). The knapsack problem is as follows: given a set of items, each with a value and a weight, the aim is to find a collection of items that maximises the value such that the total weight is less than or equal to some given limit. Since each item is included or not we have to solve the so-called 0-1 type knapsack problem. In our problem, the ‘items’ are the sub-populations that are saturated by treatment and the values and weights of each sub-population, can be read off from Eqs. (22) and (23) as

24 $$\begin{aligned}&\text {Value}_i = \left( C_0^{(i)} - C_T^{(i)}\right) N_i, \end{aligned}$$

25 $$\begin{aligned}&\text {Weight}_i = k_i\gamma _C^{(i)}N_i. \end{aligned}$$

#### Knapsack Approximation

One of the standard approaches to solving the knapsack problem computationally and the one we use here is the so-called *Meet in the middle method* (Horowitz and Sartaj 1974). This algorithm is a variation on the brute force approach searching through all the possible subsets *S*. The *Meet in the middle* algorithm consists of the following steps:

Meet in the middle algorithm

1. Split the *n* sub-populations into two subsets of approximately equal size in terms of the total value, *A* and *B*.
2. Find the total weight ($\sum _i k_i\gamma _C^{(i)}N_i$) and the total value ($\sum _i(C_0^{(i)} - C_T^{(i)})N_i$) of each subset of *A* and each subset of *B*.
3. For each subset of *A*, find the subset of *B* that maximises the value with the combined weight less than the limit *M*. This can be done efficiently as follows. First sort the subsets of *B* by weight. Then remove all subsets of *B* that have higher weight but smaller value than some other subset of *B*. That is, if for two subsets of *B*, weight $(S_1)\ge \text {weight}(S_2)$ but $\text {value}(S_1)\le \text {value}(S_2)$, remove $S_1$, because it will definitely not be in the optimal selection. After this procedure, the subsets of *B* are sorted both in weight and value. To find the subset of *B* that for some given subset of *A* maximises the value while having combined weight less than *M*, we can just use binary search.

Both steps (2) and (3) require $O(n2^{n/2})$ operations and so the whole algorithm requires $O(n2^{n/2})$ operations. Computational time for a brute force approach to finding the optimal set *S* exactly is $O(n2^n)$. Therefore, the *Meet in the middle* algorithm is significantly faster than the naive brute force search, particularly when the number of sub-populations (*n*) is large. There are other algorithms for solving this type of a knapsack problem, for example algorithms based on dynamic programming (Cormen et al. 2001) that can sometimes be faster, but for our purposes here the *Meet in the middle* algorithm is sufficient.

#### Allocation of Remaining Resources

We now consider where to allocate the remaining resources. Suppose there is not enough resource to saturate any of the sub-populations. If resources are allocated to sub-population *m*, the objective function is

26 $$\begin{aligned} J(m) = \sum _i^n C_0^{(i)}N_i - \frac{1}{2} C_0^{(m)}N_m \left( 1- \sqrt{1 - \frac{4\eta _m M}{\beta _m k_m N_m \left( C_0^{(m)}\right) ^2}}\right) . \end{aligned}$$

To minimise the objective function, involves maximising a function *f*(*m*) given by,

27 $$\begin{aligned} f(m) = C_0^{(m)}N_m\left( 1 - \sqrt{1 - \frac{4\eta _m M}{\beta _m k_m N_m \left( C_0^{(m)}\right) ^2 }}\right) . \end{aligned}$$

There is no simple rule that maximises *f*(*m*). Therefore we pick the best *m* numerically, simply by running through all $m\in \lbrace 1,2,\ldots , n\rbrace$ and selecting the one that maximises *f*(*m*).

### Calculation of True Optimal Strategy

To assess how well the approximate optimal allocation strategy given by the knapsack problem performs, we compare it with the exact optimal solution. Since we have proved that the optimal solution must saturate a subset of sub-populations, we obtain the exact optimal strategy by finding the optimal saturation set *S* that minimises the objective function (19) numerically. Specifically, we use a brute force approach and scan through all the subsets $S\subset \lbrace 1,2,\ldots , n\rbrace$ which can be saturated given the resource constraint and select the one that minimises the objective function. This has computational complexity proportional to $n2^n$, however since the largest *n* we consider is 11 such an approach remains computationally tractable.

## Results

### Performance of Knapsack Approximation

**Fig. 1:**
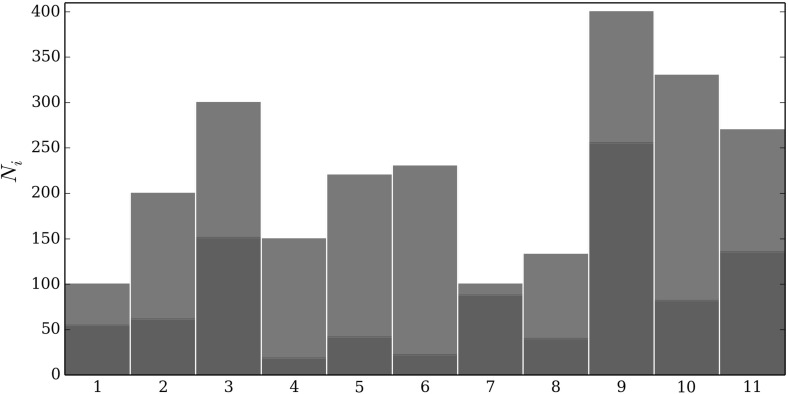
The green colour represents initial susceptible hosts and the red colour the infected ones. The columns correspond to the different populations, and the height of the columns corresponds to the population size (Color figure online)

**Fig. 2:**
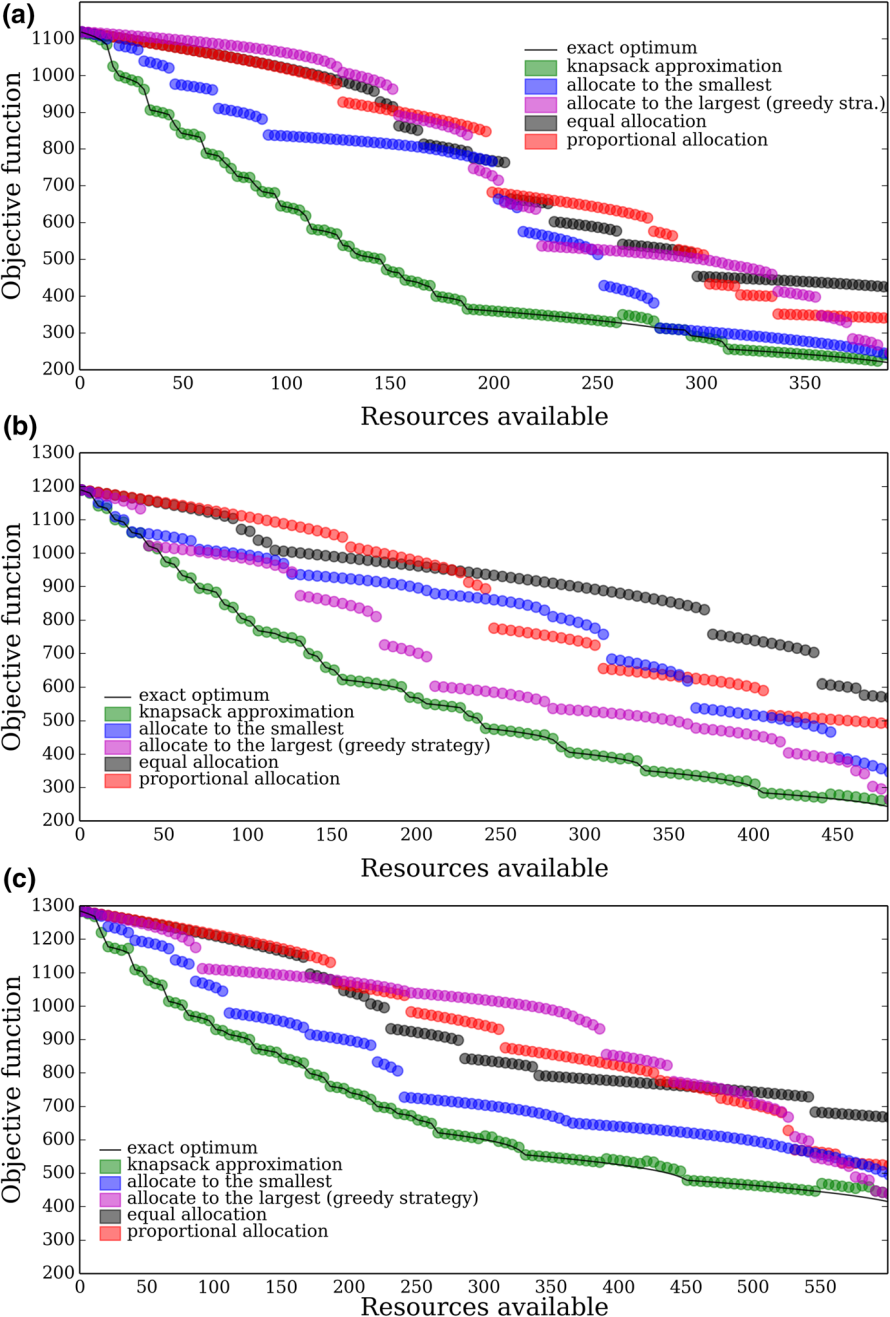
Comparison of the performance of the knapsack approximation, exact optimal calculated using a brute force approach (Sect. 2.4) and simple allocation strategies (described in Sect. 2.2) for three different realisations of the random sets of parameters $\beta _i$, $\eta _i$ and $k_i$

We initially compared the performance of the knapsack approximation, exact optimal and simple allocation strategies for many sub-populations, each with a different population structure. Since the exact optimal becomes computationally intensive to solve as the number of sub-populations increases (it is proportional to $n2^n$ where *n* is the number of sub-populations), we consider 11 sub-populations as beyond this the exact optimal is computational unfeasible to solve. The population size and initial level of infection of each sub-population are given in Fig. 1. The performance of each allocation scheme was tested on three different randomly generated combinations of parameters. More specifically a given parameter set was generated as follows: for sub-population *i* the infection rate ($\beta _i$) was chosen uniformly from the interval (1.5, 2.5), the treatment rate ($\eta _i$) was chosen uniformly from the interval (0.5, 1.1) and the cost of treating one host ($k_i$) was chosen uniformly from the interval (1, 1.5). Intervals were chosen to be in realistic ranges, and lie within the region used in previous studies Rowthorn et al. (2009); Ndeffo Mbah and Gilligan (2010). Parameter values are chosen randomly within these intervals to ensure a representative range of values from the parameter space. In this way we obtain a set of values for each sub-population that describes the disease dynamics and economic costs for that sub-population and these values taken together across all 11 sub-populations comprise a single parameter set. Figure 2 shows the performance of the allocation strategies, in terms of the value of the objective function (*J*), for three example combinations of randomly chosen parameter values as a function of the resource limit (*M*).

The knapsack approximation performed remarkably well for all three different parameter sets (Fig. 2). It only noticeably misses the optimal solution (black line) for a small range of resource limit values under the parameter regime in Fig. 2a, b, with an $8\%$ error in the knapsack approximation compared with the exact optimum. In comparison all four simple strategies perform significantly worse than the optimal strategy. Indeed the allocate to the largest strategy does worst of all for a wide range of resource limits (Fig. 2a, c). In particular, the allocate to the largest often performs worse than the equal and proportional allocation strategies. This is surprising because the allocate to the largest strategy, like the optimal strategy, saturates some subset of sub-populations while under the equal and proportional allocation strategies it is possible that no sub-populations are saturated. The poor performance of the allocate to the largest strategy therefore suggests that the choice of which sub-populations to saturate is very important, and picking the ‘wrong’ sub-populations could mean that a socially equitable solution is preferable, even if no sub-populations are saturated.

These results suggest that the knapsack approximation performs well when there are many populations. However, due to the computational cost of solving the exact optimum when there are 11 sub-populations, this limits the number of parameter sets for which the knapsack approximation can be tested. Therefore, the knapsack approximation was also tested against the exact optimum when there are just three sub-populations, $n=3$. In the case of $n=3$ the exact optimum is relatively fast to compute, allowing us to compare the knapsack and exact optimum for 50, 000 different parameter sets. These 50, 000 parameter sets were generated as follows. The parameter values in the first sub-population were kept fixed across all 50, 000 parameter sets with $\beta _1=2$, $\eta _1=1.2$, $k_1 = 1$, $N_1=100$ and the initial condition set to the endemic equilibrium. For the other two sub-populations, 50, 000 different parameter sets are generated randomly. More specifically a given parameter set for population *i* ($i=2,3$) is generated as follows: $\beta _i$ is uniform on (2,3), $\eta _i=\beta _i- 1 + r$ where *r* is a uniformly distributed random number on (0,0.5), $k_i$ is uniform on (1,1.5) and the population size ($N_i$) is uniform on (100, 1000). We note that $\eta _i$ is set to be a function of $\beta _i$ to ensure that $\eta _i>(\beta _i-1)/2$. In this way we obtain 50, 000 unique parameter sets that describe the population structure, epidemiological dynamics and economic costs for the 3 different sub-populations. Parameter values are chosen at random to ensure that we test the knapsack approximation over a wide range of parameter space.

The computational cost of solving the exact optimum is proportional to $n2^n$ (*n* is the number of sub-populations) and so solving the exact optimum when $n=11$ involves significant computational time. This limits the number of different parameter sets that could be used to test the knapsack approximation for the population structure given in Fig. 1 as the resource limit is varied. Therefore, the knapsack approximation was also tested against the exact optimum across a wide range of different parameter sets when there are just three sub-populations, $n=3$. The parameter values for the first sub-population were kept fixed with $\beta =2$, $\eta =1.2$, $k_1 = 1$, $N_1=100$ and the initial condition endemic. The parameters for the remaining two sub-populations were selected at random in the following manner. $\beta$ is uniform on (2,3), $\eta$ is $\beta - 1 + r$ where *r* is a uniformly distributed random number on (0,0.5) (this is to ensure that $\eta >(\beta -1)/2$), *k* is uniform on (1,1.5) and the population size ($N_2$ and $N_3$) is uniform on 100, 1000. We considered 50,000 different sets of parameter combinations and for each the largest error as the resource limit is varied was computed, hereafter referred to as the *worst-case error*.

Across the 50,000 different parameter sets tested, the mean worst-case error was $0.28\%$ and the standard deviation was $1.19\%$. In particular, in 91% of cases the worst-case error was 0. Furthermore, large values of the error were rare with only 1.2% of the parameter sets having a worst-case error larger than $6\%$ and the maximum error out of all 50, 000 different parameter sets was only 16.7%. This provides further support that the knapsack approximation closely approximates the exact optimum over a large range of parameter space.

**Fig. 3:**
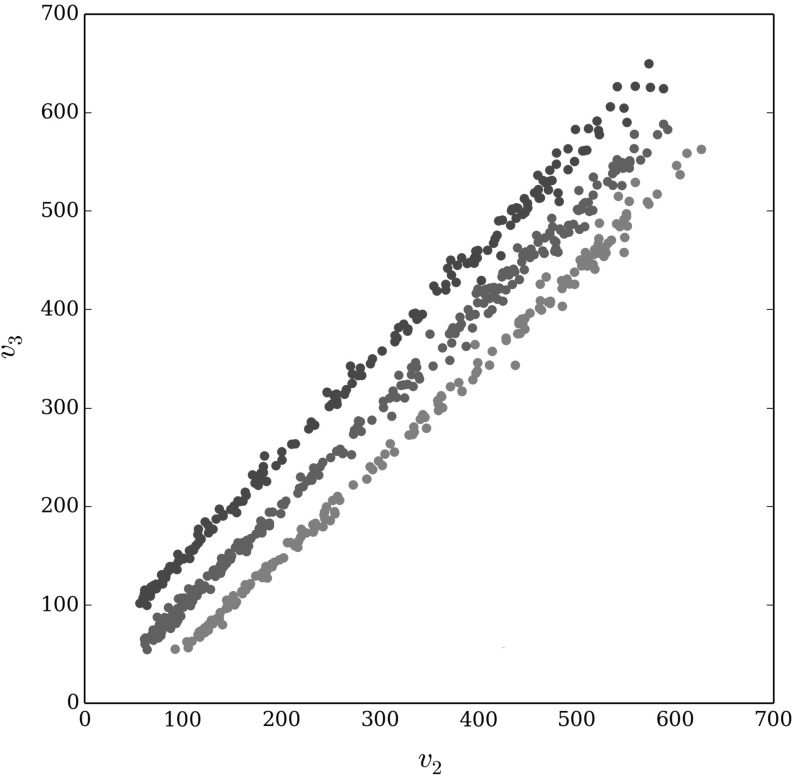
Scatter plot of the values for the two sub-populations ($v_2$ and $v_3$) whose parameters were randomly varied in the 50, 000 different parameter sets tested, for the cases where the worst-case error was higher than 6%. In particular, each point corresponds to a specific parameter set where the worst-case error was higher than 6%. Points are colour coded depending which of the three different lines they lie on. The three lines are $v_3 = v_2$ and $v_3 = v_2 \pm v_1$, where $v_1$ is the value of the sub-population whose parameter values are kept fixed for the 50,000 different sets of parameter combinations tested (Color figure online)

To understand the conditions under which the error in the knapsack is large (greater than $6\%$) we consider the relationship between the knapsack values (Eq. 24) of the two sub-populations whose parameter values are varied across the 50,000 different parameter sets tested, Fig. 3. When the worst-case error is large, the knapsack values in the two sub-populations are almost perfectly correlated with each other and in fact lie along one of three lines, $v_3 = v_2$ and $v_3 = v_2 \pm v_1$, where $v_1$ is the value of the sub-population whose parameter values are kept fixed for the 50,000 different parameter combinations tested (Fig. 3). These results suggest that the error between the knapsack approximation and the exact optimum will be largest when the knapsack values of one of the sub-populations are close to the knapsack value of one of the remaining sub-populations, or close to the sum of knapsack values of a subset of the remaining sub-populations. In these cases, the discrepancy in the total value from saturating different subsets of the sub-populations will be smaller, making it difficult for the knapsack approximation to determine which set of sub-populations it is best to saturate. Unlike the exact optimal solution, the knapsack ignores the interdependency between the set of sub-populations to saturate and where the resources remaining after no more sub-populations can be saturated should be allocated. The results here suggest that when the value of saturating sub-populations is highly correlated with each other, the interdependency between the choice of sub-populations to saturate and the allocation of the remaining resources becomes more important in determining the optimal allocation of resources. However, we note that while in these situations the knapsack approximation seems to perform less well, the maximum error between the knapsack approximation and exact optimum that we found was still only 16.7%. Furthermore, the worst-case error between the knapsack approximation and exact optimum was greater than 6% in only a very small number of cases (about 1.2% of cases tested here) suggesting that the knapsack approximation is very close to the optimal in the vase majority of cases.

We have shown that the knapsack approximation closely approximates the exact optimum. However, unlike the exact optimum, it provides analytic insight into the way to choose which sub-populations to saturate. This is since we have derived an analytic formula for the values and weights in the knapsack approximation in terms of the model parameters. In the next two subsections we use the knapsack approximation to gain insight into the characteristics that are important to capture within an allocation strategy.

### Insight into Choosing Sub-populations to Saturate Using the Knapsack Approximation

The knapsack approximation associates a value and a weight to saturating each sub-population. The value of the sub-population represents the gain that is achieved from saturation (see Eq. 24). It depends on the size of the sub-population $N_i$, as well as the disease characteristics that are captured in the endemic equilibrium with and without treatment. The knapsack approximation shows that saturating larger sub-populations (greater $N_i$) is more advantageous. It also shows that diseases for which treatment leads to a greater reduction in the endemic equilibrium (greater $C_0^{(i)}-C_T^{(i)}$) also lead to bigger gains in the objective function. However, unlike the simple strategies (as described in Sect. 2.2), the knapsack approximation also takes into account the cost of saturating a sub-population (Eq. 25), and in particular how this depends on initial conditions when $\eta _i>(\beta _i-1)/2$.

**Fig. 4:**
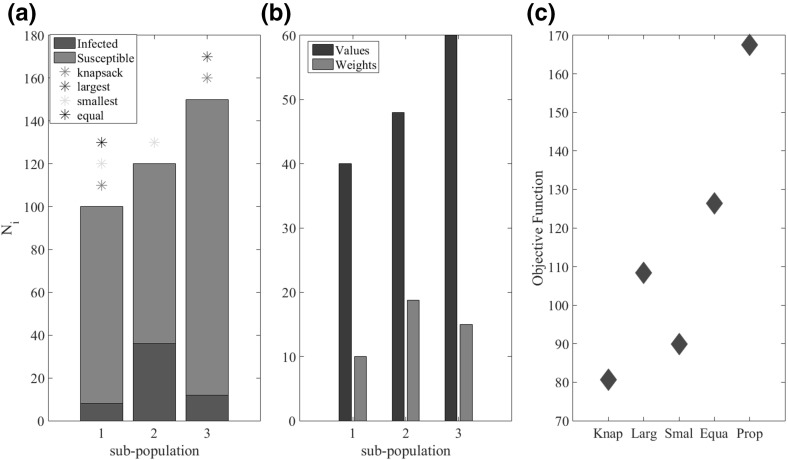
**a** Initial number of infected individuals (green) and susceptible individuals (red) for the three different sub-populations. The stars above the bars show which populations are saturated under the different allocation schemes. Note that the proportional allocation strategy does not saturate any sub-population for this example and so there are no stars corresponding to this scheme. **b** The value (blue) and weight (red) of the knapsack approximation (Eq. 24 and 25) for each sub-population. **c** Performance of the knapsack and four simple allocation strategies, in terms of the objective function, for the simple three sub-populations example. The parameter values for this example are as follows: maximum amount of resource is $M=30$ units, the sub-population sizes and initial levels of infection are $N_1=100$, $I_0^{1}=0.08$, $N_2=120$, $I_0^{2}=0.3$, $N_3=150$, $I_0^{3}=0.08$, the transmission rate for all sub-populations is $\beta _{1, 2, 3}=2$ and $\eta _{1, 2, 3}=0.8$ and the cost for all sub-populations is $k_{1, 2, 3}=1$ (Color figure online)

To understand the characteristics of the optimal allocation strategy that the knapsack captures, and in particular those that are missing from the simple strategies we consider an example of three sub-populations that are identical except for their size and initial level of infection. The size of each sub-population, along with the initial level of infection, is shown in Fig. 4a. The values of the epidemiological parameters, $\beta$ and $\eta$ are given in Table 1. The stars in Fig. 4a show which sub-populations are saturated under the knapsack approach and the other four simple strategies. Note that no sub-populations are saturated under the proportional allocation strategy and so no stars are shown for this strategy (Fig. 4a). The performance of each strategy, in terms of the objective function, is given in Fig. 4c. We conclude that since the aim is to minimise the objective function it is clear that the knapsack approach is the best allocation strategy.

Considering the sub-populations that are, and are not saturated by the different allocation strategies provides insight into the knapsack approximations superior performance. Both the knapsack and the allocate to the largest strategies saturate the largest sub-population (sub-population 3; Fig. 4a). This is because the large size of sub-population 3 means that there are potentially large gains to be made from focusing treatment in this sub-population. However, the allocate to the largest strategy also saturates the second largest sub-population (sub-population 2) while the knapsack approximation instead focuses treatment on the smallest sub-population (sub-population 1). While the value of sub-population 2 is larger than sub-population 1 (Fig. 4b), the higher initial level of infection in sub-population 2 means that a greater amount of resources need to be allocated in order to saturate sub-population 2. That is, the cost of saturating sub-population 2 is greater than for sub-population 1 (Fig. 4b). By focusing resources on sub-population 1 instead, the knapsack allocation is able to saturate two sub-populations, while the allocate to the largest is only able to saturate sub-population 3 as there are not enough resources remaining to saturate sub-population 2 as well. On the other-hand, the allocate to the smallest, like the knapsack approximation, is able to saturate two sub-populations, namely sub-populations 1 and 2. However, as with the allocate to the largest strategy, the allocate to the smallest ignores the fact that saturating sub-population 2 is much more costly, and less valuable, than saturating sub-population 3 (Fig. 4b) since the initial level of infection is so high in sub-population 2. This simple example illustrates that the knapsack performs so well because it accounts for both the value and cost of saturating a sub-population with treatment. In particular, the cost of saturation is important since the more resources that are used treating one sub-population, the fewer resources there are left to treat the remaining sub-populations. This indirect coupling that arises due to the shared limited resources is captured by the knapsack approximation but is ignored by all the simple strategies.

### The allocation of Resources Across Identical Sub-populations

**Fig. 5:**
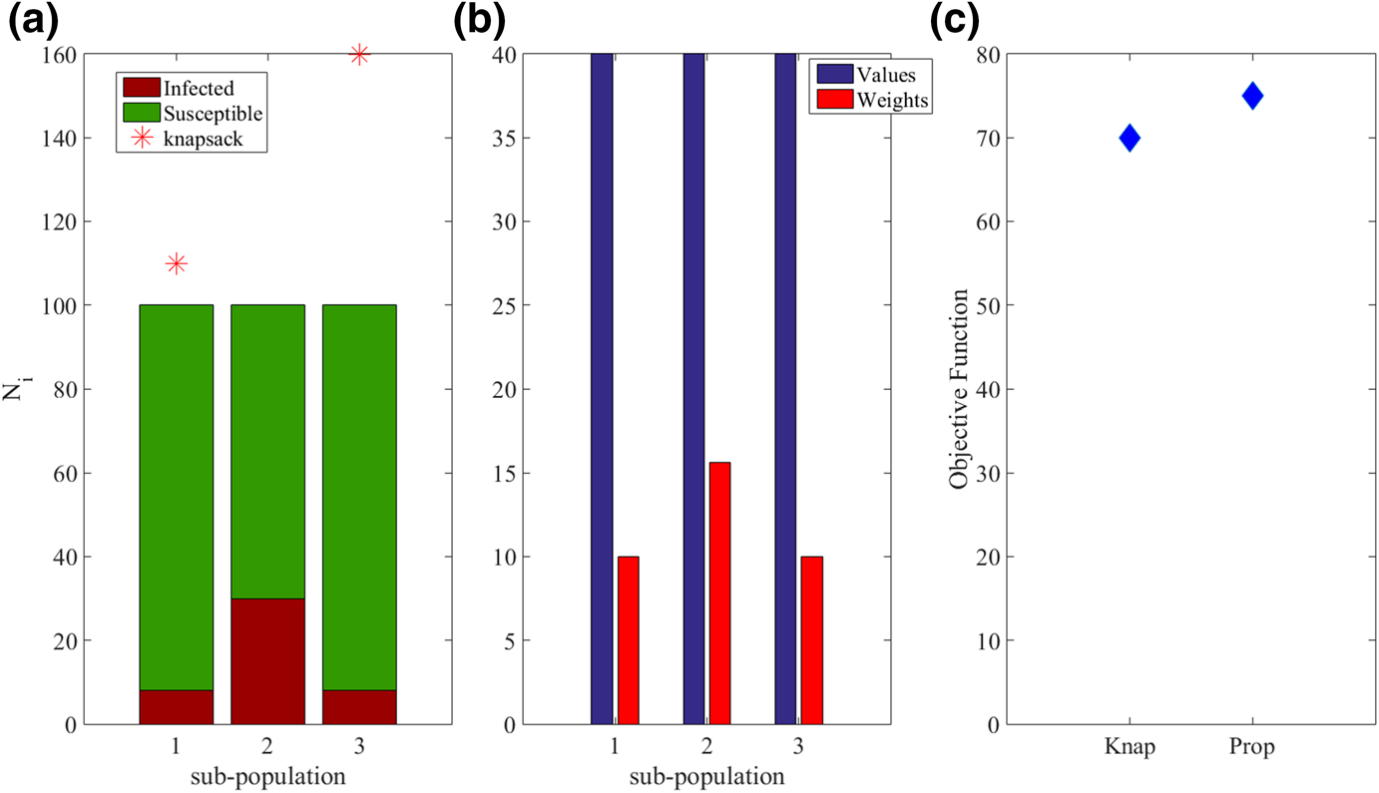
**a** Initial number of infected individuals (blue) and susceptible individuals (yellow) for the three different populations. The stars above the bars show which populations are saturated under the knapsack allocation schemes. Note that the proportional (which is equivalent to the equal allocation strategy when the sub-populations are identical) allocation strategy does not saturate any population for this example and so there are no stars corresponding to this scheme. **b**The value (blue) and weight (red) of the knapsack approximation (Eq. 24 and 25) for each sub-population. **c** Performance of the knapsack and proportional allocation strategies, in terms of the objective function. The parameter values for this example are as follows: maximum amount of resource is $M=20$ units, the sub-population sizes and initial levels of infection are $N_{1,2,3}=100$, $I_0^{1}=0.08$, $I_0^{2}=0.3$, $I_0^{3}=0.08$, the transmission rate for all sub-populations is $\beta _{1, 2, 3}=2$ and $\eta _{1, 2, 3}=0.8$ and the cost for all populations is $k_{1, 2, 3}=1$ (Color figure online)

We consider a further simplification to the 3 sub-population example in Fig. 4 such that the sizes of all 3 sub-populations are identical and so the only differences between the sub-populations are the initial levels of infection. The size of each sub-population, along with the initial level of infection is shown in Fig. 5a. Under the knapsack allocation strategy, which we have shown is approximately optimal, the aim is to maximise the value obtained from the sub-populations saturated with treatment, subject to the constraint on the resources available for treatment. Since the size, value and epidemiological parameters of the sub-populations are identical, the value of saturating a sub-population within the knapsack problem, given by Eq. (24), is the same for all sub-populations. Therefore, the more sub-populations saturated the greater the value for a given allocation strategy. The only difference between sub-populations is in the initial levels of infection and therefore the minimum amounts of treatment ($\gamma _{i}$) required to saturate each sub-population. In terms of the knapsack approximation, this means that the sub-populations have different weights given by Eq. (25) (Fig. 5b); sub-populations with smaller initial levels of infection have lower weight while higher initial prevalence leads to the sub-population having greater weight. Saturating sub-populations with fewer initial infected individuals (lower initial prevalence), a greater number of sub-populations can be saturated with treatment for a given amount of resource. Therefore, the application of the knapsack approximation suggests that, when sub-populations are identical except for the initial levels of infection, the best strategy is preferentially to treat the sub-populations with the lowest prevalence of infected individuals. This counterintuitive result is similar to what Rowthorn et al. (2009) find in the case of two identical (interacting) populations.

## Discussion

In this paper we have investigated the allocation of limited resources across *n* separate sub-populations to treat infected individuals for SIS-type epidemics. The shared resource pool introduces an effective interaction between the sub-populations, because allocating resource to one of them implies there will be less of the resource left for the others. This has the effect of anti-correlating the levels of infection in the different sub-populations. We assume that once allocated for disease control the resource cannot be reallocated later, meaning that the allocation strategy must take into account the long-term behaviour of the epidemic.

Using understanding of the long-term dynamics of the system, we have shown that the optimal allocation strategy involves saturating a subset of sub-populations with treatment, while other sub-populations receive none. This is similar to the findings for the two population case where resources should be focused within the population that is more/less infected depending on the epidemiological model and parameters, (Rowthorn et al. 2009; Ndeffo Mbah and Gilligan 2011). Characterising the full optimal allocation problem for $n\ge 2$ sub-populations is complicated by the dependence of where the remaining resources are allocated on the subset of sub-populations that are saturated with treatment. Therefore, it is not possible to characterise the full optimal allocation strategy analytically.

By ignoring where the remaining resources are allocated we are able to approximate the full optimisation problem in a way that provides greater insight into how resources should be allocated between the different sub-populations. We term this approximately optimal strategy the knapsack approximation due to its similarity to the knapsack problem from computer science (Kellerer et al. 2004). Formulating the optimisation problem in this way associates a value and a weight to each sub-population. The value of a sub-population is the gain expected from saturation while the weight is the cost of doing so. A key advantage of the knapsack approximation is that it provides formulae for the values and weights of each sub-population in terms of the epidemiological parameters. We showed that the knapsack closely approximates the exact optimum allocation strategy (obtained by brute force method) for a wide range of different parameter sets. Indeed in the worst cases, the knapsack approximation under performs the exact optimum by only $10\%$. Furthermore, the knapsack is more computationally efficient than the exact optimum, particularly when the number of sub-populations (*n*) is large.

We showed that the knapsack outperforms a number of simple strategies. The simple strategies either allocate a proportion of resources to each sub-population in a socially equitable way so everyone who is infected has an equal chance of receiving treatment or focus resources on sub-populations sequentially in a way that leads to the largest/smallest gain in the objective function. In particular, the simple strategies are chosen since they do not require any computation to determine where resources should be allocated. The knapsack performs better than the simple strategies because it captures two important aspects of the exact optimal solution which the simple strategies do not:

1. The knapsack captures the indirect coupling between sub-populations that arises due to the shared pool of resources. This is because it accounts for both the value gained in saturating a sub-population, as well as the cost (weight) of saturation and therefore the reduction in resources left over for the remaining sub-populations.
2. The knapsack captures the dependence of the optimal solution on the initial levels of infection in each sub-population. This arises due to the dependence of the minimum amount of resources needed to saturate a sub-population on the initial conditions.

We note that even though there is no direct interaction between sub-populations, the superior performance of the knapsack approximation shows that the indirect interaction between sub-populations, via the shared pool of limited resources, plays a significant role in the optimal allocation of resources. The knapsack strategy therefore provides an approximately optimal allocation strategy that is more intuitive and computationally efficient to compute than the exact optimum (particularly when the number of sub-populations is large) but performs significantly better than other straightforward allocation strategies.

The superior performance of the knapsack approximation compared with other allocation strategies that involve saturating sub-populations, such as the ‘allocate to the largest’ and ‘allocate to the smallest’ strategies, shows that the choice of which sub-populations to saturate is highly important. Indeed a strategy that saturates the ‘wrong’ sub-populations can actually do worse than a more equitable allocation strategy. This is similar to the findings of Rowthorn et al. (2009) for an SIS epidemic who found that a strategy that focuses resources on the population with the highest prevalence performs worst of all, in the case of two identical connected populations.

A key goal of this work was to extend the results from Rowthorn et al. (2009), who consider the allocation of resources between two interacting sub-populations for an SIS-type infection over a finite time horizon, to the general problem of *n* heterogeneous sub-populations. Due to the challenges of this n-dimensional problem we made two simplifications; we assumed no interaction between sub-populations and we consider the allocation of resources over a long time horizon ($T\rightarrow \infty$). Therefore, it is difficult to compare our findings directly with those of Rowthorn et al. (2009). However, by considering 3 sub-populations that are identical except for the initial levels of infection, the similarities between the results we obtain from the knapsack approximation and those reported in (Rowthorn et al. 2009) are evident. We find that in this case, the knapsack strategy involves focusing treatment on the sub-populations with the lowest level of initial infection, since these sub-populations have the lowest costs associated with saturation. This is the same as for Rowthorn et al. (2009) who find that in the case of two sub-populations, limited treatment resources should be focused in the least infected region, where there are the most susceptibles. Therefore, the results from the knapsack approximation suggest that the results from (Rowthorn et al. 2009) hold in the general n-dimensional problem for sub-populations whose size and epidemiological behaviour is identical. However, when the sub-populations are heterogeneous in terms of their size and/or epidemic dynamics it is a combination of population size, costs of infection, epidemiological parameters and initial levels of infection that determine where scarce resources should be concentrated. That may not necessarily be to the least infected sub-populations.

The objective function in Eq. (2) we use is a special case of a more general, commonly used objective function (Rowthorn et al. 2009; Ndeffo Mbah and Gilligan 2011) defined as the average cost of infection over a long-term horizon $T\rightarrow \infty$, considering a short time horizon (Zaric and Brandeau 2001a) could significantly change the results. We do not explicitly consider any discounting of the cost of future infections (Forster and Gilligan 2007). Generally, due to how our objective function is defined, an exponential discount factor $e^{-rt}$ would only multiply the objective function by a constant and thus not affect the analysis in any way.

The situations we consider here are intentionally simplified with an SIS model, in order to gain insight into the optimal allocation. Our assumptions are therefore deliberately restrictive in order to make progress. We briefly consider the implications of the assumptions below and the options for dealing with more general and realistic epidemic scenarios. We have considered epidemics of SIS-type, and so individuals can be re-infected which is typical of many diseases such as Chlamydia (Turner et al. 2006). However, for many diseases re-infection is preceded by a period of temporary immunity and so an SIRS-type (susceptible–infected–recovered–susceptible) is more appropriate. The addition of a removed class can significantly impact the optimal allocation strategy, (Ndeffo Mbah and Gilligan 2011); therefore, it is important to consider the extension of our findings to SIRS-type infections. This is not, however, straightforward since the dynamics of the SIRS model with an economic constraint on treatment are complex. In particular the long-term dynamics involve limit cycles (Vyska and Gilligan 2016). This means it is difficult to obtain a formula for the minimum amount of treatment required to saturate the population in the weights for the knapsack problem.

In this paper we assumed that sub-populations do not interact. While this is applicable to systems where each sub-population represents a distinct pathogen threat to a given species, or when there is negligible contact between the two groups (e.g. this is typically the assumption when considering the spread of blood-borne diseases within injecting drug users and non-injecting drug users) in many systems interactions between populations are important in contributing to the invasion and persistence of a pathogen (Ferguson et al. 2001; Stacey et al. 2004; Dye and Gay 2003). For the case of two identical sub-populations, Rowthorn et al. (2009) show that interactions between populations can lead to non-intuitive allocation strategies. Therefore, an important extension to the work presented here would be to derive a simple heuristic to determine the approximately optimal allocation of limited resources amongst *n* interconnected sub-populations. Extending the approach taken here to include coupling between sub-populations is, however, not straightforward. Determining the form of the optimal allocation of resources relies on the ability to fully characterise the equilibrium behaviour of the system. Relaxing the assumption of no coupling between sub-populations means that we are no longer able to determine the equilibrium behaviour for the general problem of *n* sub-populations. Therefore, our approach does not generalise easily to the problem of *n* interconnected sub-populations, and more computational methods are most likely needed to determine the optimal allocation strategy in this situation. The challenge is that in moving to more computational based methods to determine the optimal strategy we loose the intuitive insight that the analytic approach taken here provides us, with the solution to the problem being more or less a black box.

We assumed that resources are allocated at the beginning and cannot be reallocated later. While this assumption is applicable to situations where reallocation of resources may be very expensive, it ignores the fact that we may need fewer resources to maintain the endemic treatment equilibrium than are needed to reach this state initially. Therefore, surplus resources could more efficiently be used by reallocation to another population. Another natural extension to our current work would thus be to consider how allowing for reallocation of resources alters the optimal strategy. Indeed in the case of two identical populations the optimal allocation strategy for both the SIS model (Rowthorn et al. 2009) and the SIRS model (Ndeffo Mbah and Gilligan 2011) involves the continual reallocation of resources.

In this paper we consider the optimal allocation of a treatment that increases the recovery period of an individual, for example application of a fungicide or administering antibiotics. Other control measures, such as antivirals which reduce viral load or improving condom use in the case of sexually transmitted diseases, reduce the transmission rate of infection. Brandeau et al. (2003) consider such a case and find that the optimal strategy depends on many factors including the size of each sub-population, the state of the epidemic in each sub-population before resources are allocated and the effectiveness of the control program. The approach taken here could be extended to obtain an approximately optimal allocation strategy for a control which reduced the transmission rate instead of increasing the recovery rate. Since we include the economic constraint directly into the model this would involve re-framing of the problem and re-doing the long time analysis. It is therefore beyond the scope of this paper and we leave such analysis for future work.

Optimal control theory provides a powerful tool to combine epidemiological dynamics with economic factors to determine the optimal allocation of resources, which is of particular importance when resources are limited. However, the solution to such problems can often be complex to implement when for example involving multiple switching times. Previous approaches to optimal control theory may provide little intuitive insight into how epidemic spread and economic constraints impact the way in which scarce resources can be most efficiently deployed. The advantage of the approach taken here is that it provides insight into the form of the optimal solution which allows us to obtain an approximate heuristic that is simple to interpret and implement. Such simple heuristics are important in the translation of theoretical results to application by decision makers for current and future pathogen threats.

## Appendix

### Dynamic Behaviour of SIS Model

We examine the long-term behaviour of the level of infection in each population in order to gain understanding into the form of the optimal solution. In each population, the dynamics of the level of infection is governed by the following equation

28 $$\begin{aligned} \frac{dI}{dt} = \beta I(1-I) - I - \eta \min (I, \gamma ). \end{aligned}$$

Note that we have dropped the subscript *i* for brevity. $\beta$ is the transmission rate, $\eta$ is the increase in recovery rate dues to treatment and $\gamma$ is the proportion of hosts that can be treated at any given time and so this parameter captures the limited treatment resources available.

In the absence of treatment, Eq. (28) has a single nonzero fixed point,

29 $$\begin{aligned} C_0=1-\frac{1}{\beta }. \end{aligned}$$

This fixed point exists and is stable providing $\beta >1$.

When there is sufficient treatment, $I<\gamma$, the fixed points are solutions to

30 $$\begin{aligned} 0 = \beta I(1-I) - (1+\eta )I. \end{aligned}$$

Equation (30) has one nonzero solution, $C_T$, which is the endemic equilibrium proportion of infected hosts providing there is full treatment and it is given by

31 $$\begin{aligned} C_T = 1 - \frac{1 + \eta }{\beta }. \end{aligned}$$

For $C_T$ to exist we require $C_T\ge 0$, but we also need that

32 $$\begin{aligned} C_T<\gamma , \end{aligned}$$

otherwise it would be outside the relevant interval.

If there is insufficient resource to treat all infected individuals, $I > \gamma$, the fixed points are then solutions to the equation

33 $$\begin{aligned} 0 = \beta I(1-I) - I - \eta \gamma . \end{aligned}$$

This is a quadratic equation with two fixed points, *A* and *B*, given by

34 $$\begin{aligned}&I_A = \frac{1}{2}\left( C_0 - \sqrt{C_0^2 - \frac{4\eta \gamma }{\beta }}\right) , \end{aligned}$$

35 $$\begin{aligned}&I_B = \frac{1}{2}\left( C_0 + \sqrt{C_0^2 - \frac{4\eta \gamma }{\beta }}\right) . \end{aligned}$$

*A* is always unstable, whereas *B* is always stable. This is because in the quadratic in Eq. (33), the coefficient in front of $I^2$ is negative. Therefore perturbing the system slightly from the root *B* in the positive (negative) direction results in a negative (positive) derivative and vice-versa for *A*.

For these points to exist $C_0^2 > 4\eta \gamma /\beta$, otherwise *A* and *B* would be imaginary. This condition simply puts a restriction on $\gamma$:

36 $$\begin{aligned} \gamma < \frac{\beta C_0^2}{4\eta }\equiv \gamma _\text {max}. \end{aligned}$$

An additional constraint occurs, $I_{A,B}>\gamma$, otherwise these points would lie outside the relevant interval. For $I_B > \gamma$ we need that,

37 $$\begin{aligned} 2\gamma - C_0 < \sqrt{C_0^2 - \frac{4\eta \gamma }{\beta }}, \end{aligned}$$

which is satisfied automatically whenever $\gamma < C_0/2$. If $\gamma \ge C_0/2$ is squared the inequality to obtain

38 $$\begin{aligned}&\gamma - C_0 < -\frac{\eta }{\beta } \end{aligned}$$

39 $$\begin{aligned}&\iff \quad \gamma < C_T. \end{aligned}$$

In other words, for $I_B > \gamma$ we need

40 $$\begin{aligned} \gamma <\max (C_0/2, C_T). \end{aligned}$$

Therefore, $I_{B}$ exists providing conditions 36 and 40 are met.

When both $C_{T}$ and *B* coexist, *A* must exist as in a one-dimensional system we cannot have two stable fixed points without an unstable one in between (Strogatz 2014). Conversely, when *A* exists, both $C_{T}$ and *B* must exist (although $C_{T}$ could be at 0). This means that the existence of *A* is equivalent to the simultaneous existence of $C_{T}$ and *B*. Putting together conditions (32), (36) and (40) gives that *C* exists whenever $\gamma$ satisfies both

41 $$\begin{aligned}&C_T< \gamma < C_0/2, \end{aligned}$$

42 $$\begin{aligned}&\gamma < \gamma _\text {max}. \end{aligned}$$

It follows that *A* can only exist when $C_0>2C_T$ and when $C_T < \gamma _\text {max}$. The first translates to

43 $$\begin{aligned} \eta > \frac{\beta - 1}{2} \end{aligned}$$

where we introduced a new parameter $r = \beta - 1$. The second translates to

44 $$\begin{aligned}&C_T < \frac{\beta C_0^2}{4\eta } \end{aligned}$$

45 $$\begin{aligned}&\iff \quad 4\eta (\beta - \eta - 1) < \beta ^2 C_0^2 \end{aligned}$$

46 $$\begin{aligned}&\iff \quad 4 \eta ^2 - 4\beta \eta C_0 + \beta ^2 C_0^2 > 0 \end{aligned}$$

47 $$\begin{aligned}&\iff \quad (2\eta - \beta C_0)^2 > 0. \end{aligned}$$

This is automatically satisfied and therefore we only need $\eta > (\beta - 1)/2$. It is straightforward to check that when $\eta >(\beta - 1)/2$ we have $C_0/2 > \gamma _\text {max}$ and therefore the full set of conditions equivalent to the existence of *A* is

48 $$\begin{aligned} \eta > (\beta - 1)/2\quad \text {and}\quad C_T< \gamma < \gamma _\text {max}. \end{aligned}$$

We can now summarise the fixed point behaviour of the system (28).

1. When $\eta < (\beta - 1)/2$, there is a single nonzero endemic state which is given by *B* when $\gamma <C_T$ and by $C_{T}$ (endemic equilibrium given full treatment) otherwise.
2. When $(\beta - 1)/2 < \eta$ and $\gamma$ is outside the interval $(C_T,\gamma _\text {max})$, there is only one nonzero endemic state. This endemic equilibrium is $C_{T}$ providing sufficient resources are available, namely if $\gamma >C_{T}$, otherwise the endemic equilibrium is *B*.
3. When $(\beta - 1)/2 < \eta$ and $\gamma \in (C_T,\gamma _\text {max})$, there are two endemic states ($C_{T}$ and *B*) and which one is reached depends on the initial condition. The basins of attraction of these two equilibrium states are separated by the point *A*.

### Real Symmetric Matrix with Negative Entries has Negative Eigenvalues

#### Theorem 1

Let *X* be a real, symmetric, square matrix such that $X_{ij}<0$, $\forall i,j$. Then there exists a vector *u* such that $u^T Xu < 0$, that is the matrix *X* has a negative eigenvalue.

#### Proof

Consider a vector *u* such that $u_i>0$, $\forall i$. Denote $v = Xu$. Therefore $v_i = \sum _k X_{ik}u_k < 0$, $\forall i$. This means that

49 $$\begin{aligned} u^T X u = \sum _i u_i v_i < 0. \end{aligned}$$

$$\square $$

## References

[CR1] Anderson RM, May RM (1991). Infectious diseases of humans: dynamics and control.

[CR2] Arrowsmith DK, Place CM (1992). Dynamical systems: differential equations, maps, and chaotic behaviour.

[CR3] Baker RHA, Bishop HAS, MacLeod ANP, Tuffen MG (2014). The UK plant health risk register: a tool for prioritizing actions. Bull OEPP/EPPO Bull.

[CR4] Brandeau ML, Zaric GS, Richter A (2003). Resource allocation for control of infectious diseases in multiple independent populations: beyond cost-effectiveness analysis. J Health Econ.

[CR5] Cormen TH, Leiserson CE, Rivest RL, Stein C (2001). Introduction to algorithms.

[CR6] Dye C, Gay N (2003). Modeling the SARS epidemic. Science.

[CR7] Ferguson NM, Donnelly CA, Anderson RM (2001). The foot-and-mouth epidemic in great Britain: pattern of spread and impact of interventions. Science.

[CR8] Feuerman M, Weiss H (1973). A mathematical programming model for test construction and scoring. Manag Sci.

[CR9] Forster GA, Gilligan CA (2007). Optimizing the control of disease infestations at the landscape scale. PNAS.

[CR10] Grenfell BT, Bolker BM (1998). Cities and villages: infection hierarchies in a measles metapopulation. Ecol Lett.

[CR11] Grenfell BT, Bolker BM (2000). Bubonic plague: a metapopulation model of a zoonosis. Proc R Soc B.

[CR12] Hansen E, Day T (2011). Optimal control of epidemics with limited resources. J Math Biol.

[CR13] Horowitz E, Sartaj S (1974). Computing partitions with applications to the knapsack problem. J ACM.

[CR14] Keeling MJ, Woolhouse MEJ, Shaw DJ, Matthews L, Chase-Topping M, Haydon DT, Cornell SJ, Kappey J, Wilesmith J, Grenfell BT (2001). Dynamics of the 2001 UK foot and mouth epidemic: stochastic dispersal in a heterogeneous landscape. Science.

[CR15] Kellerer H, Pferschy U, Pisinger D (2004). Knapsack problems.

[CR16] Kerr G, F WJ, Mason WL, Jinks RL, T J (2015) Building resilience into planted forests: recent experience from great britain. In: XIV World forestry congress, pp 1–10

[CR17] Kiszewski A, Johns B, Schapira A, Delacollette C, Crowell V (2007). Estimated global resources needed to attain international malaria control goals. Bull World Health Organ.

[CR18] Lipsitch M, Bergstrom CT, Levin BR (2000). The epidemiology of antibiotic resistance in hospitals: para- doxes and prescriptions. PNAS.

[CR19] Martello S, Toth P (1990). Knapsack problems: algorithms and computer implementations.

[CR20] Masoa ED, Cockingb J, Montecchioa L (2014). Efficacy tests on commercial fungicides against ash dieback in vitro and by trunk injection. Urban For Urban Green.

[CR21] Ndeffo Mbah ML, Gilligan CA (2010). Balancing detection and eradication for control of epidemics: sudden oak death in mixed-species stands. PLoS One.

[CR22] Ndeffo Mbah ML, Gilligan CA (2011). Resource allocation for epidemic control in metapopulations. PLoS one.

[CR23] Richter A, Brandeau ML, Owens DK (1999). An analysis of optimal resource allocation for prevention of infection with human immunodeficiency virus (HIV) in injection drug users and non-users. Med Decis Mak.

[CR24] Rowthorn RE, Laxminarayan R, Gilligan CA (2009). Optimal control of epidemics in metapopulations. J R Soc Interface.

[CR25] Sheremet O, Healey JR, Quine CP, Hanley N (2017). Public preferences and willingness to pay for forest disease control in the uk. J Agric Econ.

[CR26] Skiena S (1999). Who is interested in algorithms and why?: lessons from the stony brook algorithms repository. ACM SIGACT News.

[CR27] Stacey AJ, Truscott JE, Asher MJC, Gilligan CA (2004). A model for the invasion of rhizomania in the United Kingdom: implications for control strategies. Phytopathology.

[CR28] Strogatz SH (2014). Nonlinear dynamics and chaos: with applications to physics, biology, chemistry, and engineering.

[CR29] Turner KME, Adams E, LaMontagne DS, Emmett L, Baster K, Edmunds WJ (2006). Modelling the effectiveness of chlamydia screening in England. Sex Transm Infect.

[CR30] Vyska M, Gilligan CA (2016). Complex dynamical behaviour in an epidemic model with control. Bull Mathe Biol.

[CR31] Wallinga J, Edmunds W, Kretzschmar M (1999). Perspective: human contact patterns and the spread of airborne infectious diseases. Trends Microbiol.

[CR32] Zaric GS, Brandeau ML (2001). Optimal investment in a portfolio of HIV prevention programs. Med Decis Mak Int J Soc Med Decis Mak.

[CR33] Zaric GS, Brandeau ML (2001). Resource allocation for epidemic control over short time horizons. Math Biosci.

[CR34] Zhou Y, Yang K, Zhou K, Liang Y (2014). Optimal vaccination policies for an SIR model with limited resources. Acta Biotheor.

